# Influence of Electrode Connection Tracks on Biological Cell Measurements by Impedance Spectroscopy †

**DOI:** 10.3390/s19132839

**Published:** 2019-06-26

**Authors:** Arthur Luiz Alves de Araujo, Julien Claudel, Djilali Kourtiche, Mustapha Nadi

**Affiliations:** Institut Jean Lamour, Lorraine University (CNRS—UMR 7198), 54011 Nancy, France

**Keywords:** biosensor, impedance spectroscopy, biological cell detection, coplanar electrodes

## Abstract

The limit of detection of a biological sensor is an important parameter because, when it is optimized, it allows the detection of a reduced number of biological cells and the reduction of the detection time. This parameter can be improved upon with a reduction in electrode size, but the rate of detection is similarly reduced as well. To avoid this problem, we propose a sensor matrix composed of 20 × 20 µm² coplanar square electrodes with a standard clean room manufacturing process. However, it was observed that the exposition of electrode connection tracks to the solution reduces the normalized impedance variation. In this pursuit, we propose in this paper an analysis of electrode connection tracks on the normalized impedance variation and cutoff frequencies to biological cell measurements by impedance spectroscopy. The experimental results were obtained using the E4990A Keysight impedance analyser (Keysight Technologies, Santa Rosa, CA, USA) with a frequency band ranging from 100 Hz to 12 MHz, thus allowing for good measurement accuracy. Therefore, it was found that, for the measurements between the electrodes with 9 µm of connection tracks in contact with the solution, the normalized impedance variation was from 3.7% to 4.2% for different measurements, while, for the electrodes with 40 µm of connection tracks in contact with the solution, the normalized impedance variation was from 1.8% to 2.1% for different measurements.

## 1. Introduction

The characterization of biological cells and tissues is an important way to identify pathologies and to improve disease treatment or the detection of pathogens. Knowledge of the biophysical properties of cells can provide early signals of disease or abnormal conditions in the human body. However, this characterization is usually done in a laboratory using expensive equipment and lengthy procedures [1]. Reducing these constraints is one of the main interests of new technologies that have been developed to characterize cells faster, cheaper, and more accurately; these include electrorotation [2], impedance flow cytometry [3,4], microelectrical impedance spectroscopy [5,6], and electrochemical immunosensors [7]. Thus, these technologies are valuable potential markers for identifying cancers [8], bacteria [9], toxins [10], and the nature of tissues [11].

In this way, the bioimpedance spectroscopy technique with single biological particles suspended in physiological media [12], with the use of magnetic nanobeads and screen-printed interdigitated electrodes [1,13], or with microwell arrays between transparent conducting electrodes within a microfluidic channel to deliver and extract cells [14] is able to detect and characterize cells and bacteria. Combined with microfluidics structures, impedance spectroscopy can provide a powerful tool for sorting, analysing, counting, or discriminating cells [3,15,16].

The limit of detection is the lowest number of biological cells that can be detected by a biosensor. One way to improve it is with a reduction in electrode size. Importantly though, with this, the rate of detection is also reduced as well. The use of an electrode matrix increases the rate of detection; however, the electrode connection tracks (principally for the electrodes in the centre of the matrix) promote a current leakage effect. To analyse the connection tracks’ effects on impedance spectroscopy measurements for the detection of biological cells, we propose in this paper a sensor matrix composed of four 20 × 20 µm² coplanar square electrodes spaced 20 µm apart with different lengths (9 µm and 40 µm) of electrode connection tracks exposed to the solution.

The biosensor is composed of a microchannel (1500 µm in length, 100 µm in width, and 20 µm thick) to centre the sample on the electrodes, and the measurements were taken in static to analyse the frequency band. To place the sample on the electrode and to complete the static measurements, we used a low-cost system based on Pascal’s law. 

The second section of this work presents an electrical model and the associated analytical equations and graphics of the sensor. In the third section, a simulation model using Comsol (COMSOL Multiphysics® COMSOL AB, Stockholm, Sweden) as well as graphs and discussions to analyse the influence of undesired electrode connection track effects on the normalized impedance variation and frequency band are presented. In the fourth section, the manufacturing process of the sensor is shown. In the fifth section, the measurement setup based on an autobalance bridge is described and the practical results are presented. To finish, the sixth section concludes by covering the undesired electrode connection track effects on the normalized impedance variation and on the frequency band.

## 2. Sensor Design and Electric Model

### 2.1. Electrical Model

**Figure 1:**
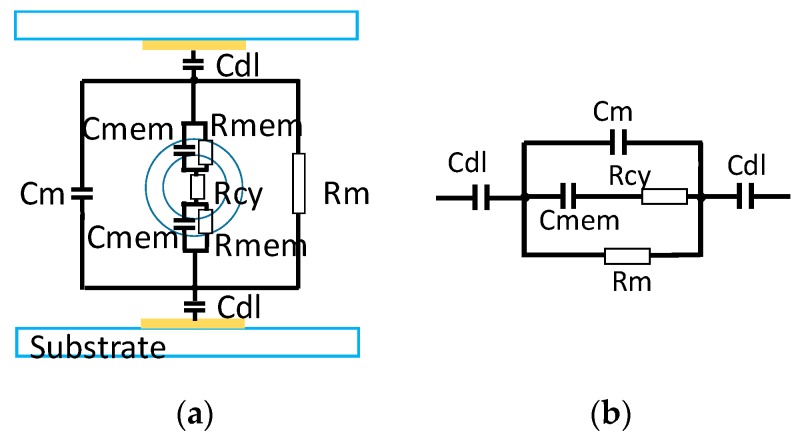
(**a**) Electrical components diagram for a cell in suspension between two identical parallel electrodes and (**b**) its electrical model.

For a cell in suspension between two identical parallel electrodes, an electrical model with an R-C series in parallel with an R-C parallel, like is shown in Figure 1b, can be used to represent the measured impedance [12]. In this model, it is not considered the substrate and electrode connection track effects like is shown in Figure 1. *C_dl_* is the capacity of the double layer effect between the electrode and the medium; R_m_ and C_m_ represent the resistance and the capacitance of the medium; and *R_cy_*, *R_mem_*, and *C_mem_* are the cytoplasmic cell resistance, the membrane cell resistance, and the membrane cell capacity, respectively. As the lipids act mainly as an insulating medium, the cell membrane resistance (*R_mem_*) is very high so that the current passes only by *C_mem_* and, thus, *R_mem_* can be neglected.

**Figure 2:**
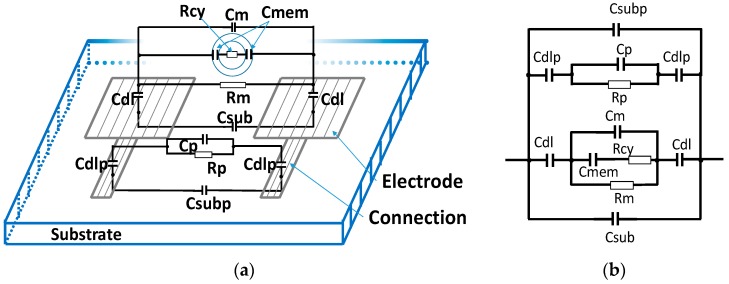
(**a**) Electrical components diagram for a pair of coplanar electrodes with a cell in the middle (**b**) and its electrical model.

In our case, presenting a cell in suspension between two identical coplanar electrodes, we developed the electrical model that is shown in Figure 2. To improve the normalized impedance variation, the electrode size can be reduced. However, this led to undesired effects due to the leakage current on the connection tracks, and the capacitive effects of the substrate (Figure 2) appeared to decrease the high cutoff frequency until short-circuiting of the impedance of the sample occured. These undesired effects are modelled in two parts: the first one by capacitors *C_sub_* and *C_subp_* for the substrate and the second one for the medium/connection track interface by resistance *R_p_* and capacitors *C_dlp_* and *C_p_*. They depend on substrate permittivity and conductivity, on the material on the connection tracks, and on the shape factor. The conductivity and the permittivity of the materials may be well-known; however, it is very difficult to model the shape factor, which is thus determined by simulation or measurements. 

Once the values of *R_m_*, *R_cy_*, *C_mem_*, and *C_m_* have been obtained by impedance measurements, intrinsic dielectric parameters can be extracted from Equations (1)–(4) [12], in which *Φ* represents the volume ratio (ratio of the cell volume/measurement volume), *K* is a shape factor depending on the geometry of the electrodes, *σ_m_* is the conductivity of the medium, *σ_cyt_* is the conductivity of the cytoplasm, *ε_m_* is the permittivity of the medium, *r* is the radius of the cell, and *C_mem,s_* is the surface capacity of the cell membrane.

(1) $$R_{m}=\frac{1}{\sigma_{m}\left(1-\frac{3\emptyset }{2}\right)K}.$$

(2) $$C_{m}=\epsilon_{m}\left(1-\frac{3\emptyset }{2}\right)K.$$

(3) $$R_{cy}=\frac{4\left(\frac{1}{2\sigma_{m}}+\frac{1}{\sigma_{cyt}}\right)}{9\emptyset K}.$$

(4) $$C_{mem}=\frac{9\emptyset rC_{mem,S}}{4}K.$$

**Figure 3:**
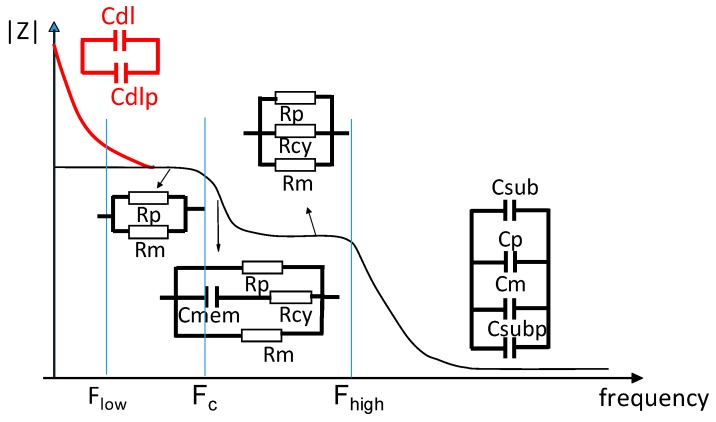
Representation of the impedance spectrum with electric models.

The resistance and the capacitor values of the electrical model are associated with the impedance spectrum obtained experimentally, as shown in Figure 3.

At low frequency, below the low cutoff frequency (*F_low_*), the capacitive effects of the double layer of electrodes (*C_dl_*) and of the double layer of connections (*C_dlp_*) are predominant, but once the frequency is increased, the *C_dl_* effect makes way for the medium resistance effect (*R_m_*) in parallel with the undesired resistance connection (*R_p_*) on the first impedance level (Figure 3) [12]. On the second impedance level, the membrane capacitance impedance (*C_mem_*) is very small and, therefore, the cytoplasmic resistance (*R_cy_*) is in parallel with the medium resistance (*R_m_*) and with the undesired resistance connection (*R_p_*). On the last impedance level, there is a medium capacitance effect (*C_m_*) in parallel with the substrate capacitance (*C_su_*_b_ and *C_subp_*) and connection capacitance (*C_p_*).

### 2.2. Normalized Impedance Variation

To calculate the normalized impedance variation, we determine the relative impedance difference between the impedance of the biosensor without the sample on the electrodes, *Z_f_*_0_, and the impedance, *Z_f_*_0*cell*_, with a sample between the electrodes, as shown in Equation (5) for a specific frequency (*F*_0_) between the cell cutoff frequency (*F_c_*) and high cutoff frequency (*F_high_*):

(5) $$\Delta \left|Z\right|=\frac{Z_{f0cell}-Z_{f0}}{Z_{f0}}.$$

In the specific frequency (*F*_0_), the impedance without a cell is equal to the medium resistance *Z_f_*_0_ *= R*_0_, while the impedance with a cell between the electrodes is equal to the parallel equivalent of the medium resistance and the cytoplasm resistance as follows: *Z_f_*_0*cell*_ = *R_m_R_cy_*/(*R_m_ + R_cy_*). From this, one can obtain the normalized impedance variation, as seen in Equation (6):

(6) $$\Delta \left|Z\right|=\frac{\frac{R_{m}R_{cy}}{R_{m}+R_{cy}}-R_{0}}{R_{0}}=\frac{R_{m}R_{cy}}{\left(R_{m}+R_{cy}\right)R_{0}}-1.$$

Using Equations (1) and (3) in Equation (6), one can obtain the impedance variation according to the intrinsic parameters of the sensor and of the biological cell (Equation (7)):

(7) $$\Delta \left|Z\right|=\frac{2+4\frac{\sigma_{m}}{\sigma_{cyt}}}{6\emptyset K\left(1-\frac{\sigma_{m}}{\sigma_{cyt}}\right)+2K\left(1+\frac{2\sigma_{m}}{\sigma_{cyt}}\right)}-1.$$

**Figure 4:**
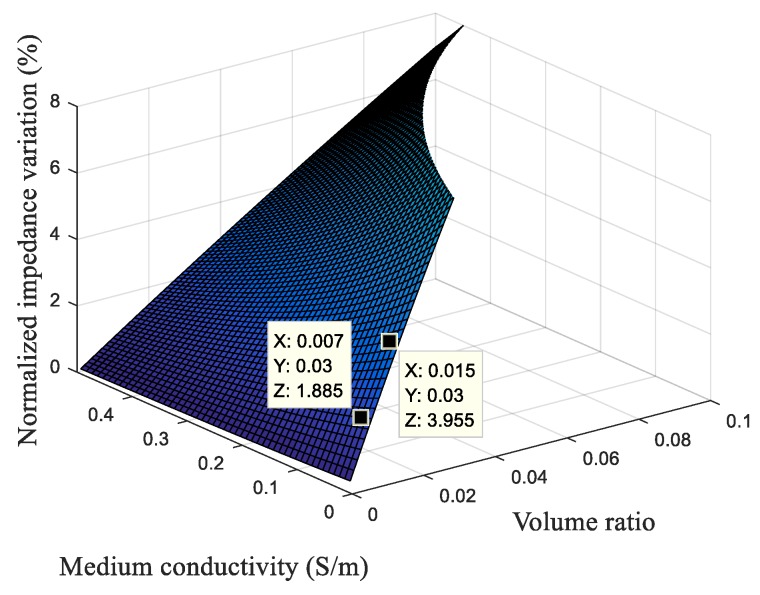
Normalized impedance variation as a function of the volume ratio (ratio of the cell volume/measurement volume—Φ) and medium conductivity for the generic parameter values.

The normalized impedance variation is proportional to the volume ratio, as one can see from Equation (7) and from the graph in Figure 4. This means that, to increase the normalized impedance variation, the volume ratio must also be increased. For this, the electrode sizes must be reduced next to the diameter of the target biological cell. We also conclude from Equation (7) and from the graph of Figure 4 that normalized impedance variation depends on the ratio between the medium conductivity and the cytoplasm conductivity.

### 2.3. Cutoff Frequency

There are three cutoff frequencies (Figure 3): the low cutoff frequency (*F_low_*), where the double-layer capacitance effect becomes negligible and the effects of the sample predominate; the cell cutoff frequency (*F_c_*), where the intrinsic cell properties can be extracted; and the high cutoff frequency (*F_high_*), where the effects of the sample become negligible and the medium capacity becomes predominant. Below *F_low_*, it is impossible to measure the intrinsic properties of the biological medium, which is also true above *F_high_*. In this way, the electrodes must be designed to ensure that *F_high_* is greater than the F_c_ and to establish a wide operating frequency band between *F_low_* and *F_high_*. From the electrical model and the impedance spectrum, it is possible to determine the low cutoff frequency *F_low_* (Equation (8)) [17], the cell cutoff frequency *F_c_* (Equation (9)), and the high cutoff frequency *F_high_* (Equation (10)):

(8) $$F_{low}=\frac{1}{2\pi R_{m}C_{dl}}.$$

(9) $$F_{c}=\frac{1}{2\pi (R_{m}+R_{cy})C_{cem}}.$$

(10) $$F_{high}=\frac{1}{2\pi R_{m}\left(C_{m}+C_{sub}+C_{p}+C_{subp}\right)}.$$

From Equation (10), one can see that, if the parasitic capacitances *C_p_*, *C_sub_*, and *C_subp_* are increased, the *F_high_* decreases and can become even lower than *F_c_*. To minimize these parasitic capacitive effects, the electrode and connection geometries must be enhanced.

**Figure 5:**
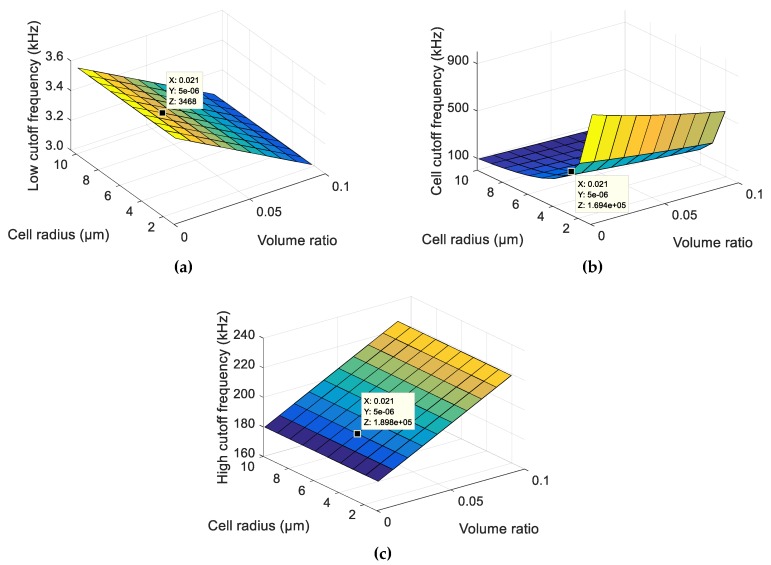
(**a**) Low cutoff frequency; (**b**) cell cutoff frequency; and (**c**) high cutoff frequency in the function of the volume ratio (ratio of the cell volume/measurement volume) and cell radius from the analytical model.

As can be seen in the graphs of Figure 5, the larger the volume ratio, the lower is the low cutoff frequency in Figure 5a and the higher is the high cutoff frequency in Figure 5c, and the change in the cell cutoff frequency is negligible in Figure 5b. With a larger volume ratio, one also has a wider frequency band with which to detect a cell. On the other hand, to have a great value of the volume ratio is very difficult because the electric field is well-dispersed and, thus, the high cutoff frequency reduces and approaches the cell cutoff frequency.

To increase the volume ratio, we must reduce the size of electrodes next to the target biological cell. For example, we can use electrodes of 20 × 20 µm² for the detection of white blood cells (around 10 µm of the radius) or electrodes of 5 × 5 µm² to detect bacteria (around 2 µm of the radius for some bacteria). On the other hand, as the size of the electrodes is reduced, the electrode connection tracks have an influence on the normalized impedance variation and on the cutoff frequencies, as shown in the simulations below. These influences of the connections are due to the undesired connection effect that becomes of the same order as the impedance of the electrodes.

## 3. Simulation

To analyse the connection effects on the normalized impedance variation and on the frequency band of the squared coplanar electrodes, the finite element method with Comsol and the electrical models described above were used.

### 3.1. Simulation Parameters

To analyse the connection effects on the cutoff frequency, the finite element method (Comsol) was used and the numerical model was divided in two parts because of the big difference between electrode connection dimensions, as follows: the channel innerelectrode connection tracks with micrometric-order dimensions and the channel outerelectrode connection tracks with millimetre-order dimensions. In this way, one obtains a parasitic capacitance (*C_plar_*) with the finite element model at the millimetre order and the values of *C_m_*, *R_m_*, and *C_p_* with a finite element model at the micrometric order. Once the values *C_plar_*, *C_p_*, *C_m_*, and *R_m_* have been obtained, MatLab (Mathworks, Natick, MA, USA) was used to plot the frequency domain curves.

**Figure 6:**
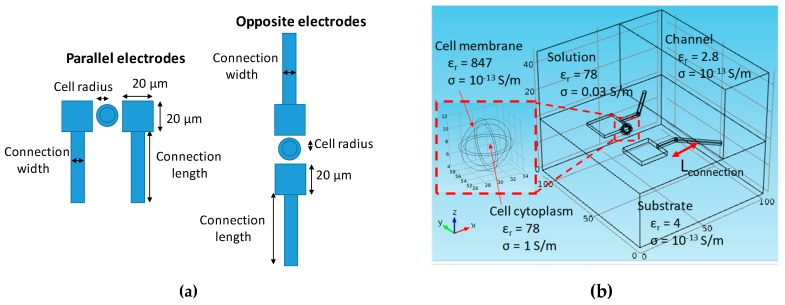
Parameters used for the finite element method simulations: (**a**) Electrode and connection dimensions and (**b**) electrical parameters of the numerical model.

Basically, cells are composed of a cytoplasm and a membrane. The cytoplasm can be considered like an ionic conductive material because it is composed of a complex mixture of substances dissolved in water. The cell membrane, composed of an insulating lipid layer of about 10 nm in thickness, can be modelled by a capacitor with a high surface capacitance (around 1 µF/m²) [3]. From this information, the biological cell can be modelled in the first approach with two concentric spheres, with the inner one being a conductor and the outer one being an insulator as shown in Figure 6b [14,18]. The use of very different dimensions (electrodes in micrometres and cell membrane in nanometres) requires a great computational power. To minimize computational costs, we have increased the thickness of the cell membrane to 0.75 μm and its electrical permittivity to have the same surface capacity (1 µF/m²).

To analyse the connection effects on the normalized impedance variation, we also used the finite element method with Comsol, but only with the micrometric part because the impedance of the channel outerelectrode connection tracks is negligible at the chosen measured frequency.

The parameters used for simulations with Comsol are shown in Figure 6. On the left, Figure 6a provides the dimensions of the electrodes and channel inner connection tracks for the parallel and opposite configurations. On the right, Figure 6b is a simulation box with the substrate, electrode, and their values of conductivity and permittivity for the parallel electrodes.

### 3.2. Simulation Results

**Figure 7:**
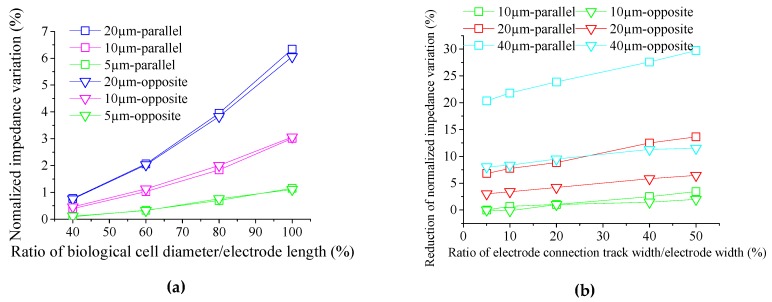
(**a**) Graph of the ratio of cell diameter/electrode width versus normalized impedance variation for three electrode sizes with the same connection track dimensions and (**b**) graph of the ratio of electrode connection track width/electrode width versus the alteration of normalized impedance variation for three different lengths of electrode connection tracks.

To analyse the influence of electrode connection tracks on the normalized impedance variation when the dimensions of the electrodes are reduced, the ratio of biological cell diameter/electrode width versus normalized impedance variation curve was established for the parallel electrodes and for the opposite electrodes (Figure 7a). To plot this graph, the size of electrode connection tracks was established as constant (10 µm in length and 2 µm in width), while the diameter of the cell varied from 40% to 100% of the electrode width for square electrodes measuring 20 µm (blue curves), 10 µm (magenta curves), and 5 µm (green curves) in length. From this graph, one can see that the normalized impedance variation is reduced when the electrode size is reduced while the electrode connection track dimension remains constant. Therefore, when the electrode dimensions are reduced, the electrode connection tracks must also be reduced; however, this is not always possible because of fabrication limitations.

The graph of Figure 7b shows the ratio of electrode connection track width/electrode width versus the reduction of the normalized impedance variation for the parallel electrodes and for the opposite electrodes with three different lengths of electrode connection tracks: 40 µm (cyan curves), 20 µm (red curves), and 10 µm (green curves). This refers to how much the normalized impedance variation is reduced when the surface of an electrode connection track increases. For a length of electrode connection track equal to twice the width of the electrodes, it is observed in Figure 7b (square cyan curve) that the normalized impedance variation decreases from 20% (electrode connection track with 1% of the size of the electrodes) until 30% (electrode connection track with 50% of the size of the electrodes). This difference of the normalized impedance variation takes as a reference the normalized impedance variation for the smallest electrode connection track with a biological cell diameter of 16 µm between the electrodes.

From theses graphs, it is observed that, when the dimensions of the electrode connection tracks are reduced, their influence on the normalized impedance variation is the same for parallel electrodes and for opposite electrodes. However, when the surface of electrode connection tracks is increased, the parallel electrodes are more influenced by the parasitic effects and their reduction of the normalized impedance variation is bigger than the opposite electrodes.

**Figure 8:**
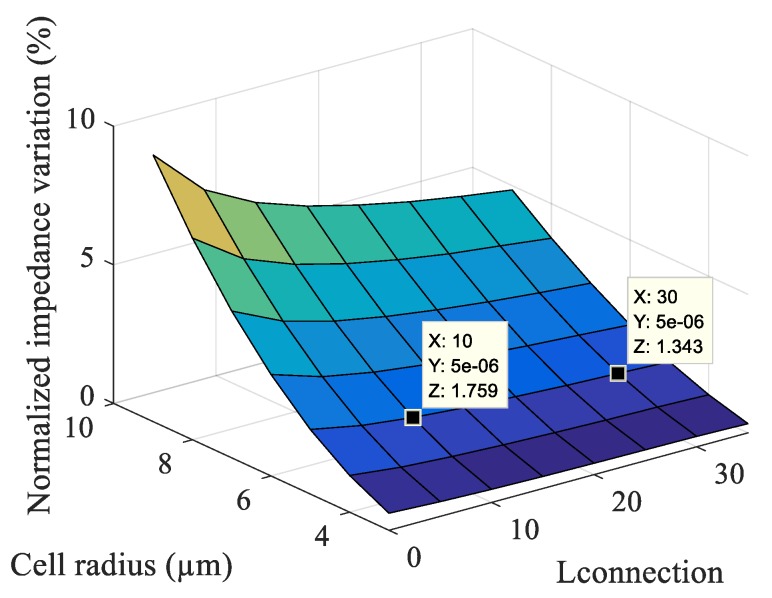
Normalized impedance variation as a function of the volume ratio and connection length.

For 20 × 20 μm² parallel electrodes and 2 μm electrode connection track width, it can be observed in the graph of Figure 8 that the bigger the electrode connection track length (*L_connection_*), the smaller is the normalized impedance variation. This graph agrees with the analytical graph (Figure 4) because, when the electrode connection track length increases, the volume of the electric field increases and, if the size of the cell does not change, the volume ratio thus decreases. Therefore, we visualize a decrease in the normalized impedance variation with a decrease of the volume ratio (from the analytical model) and with an increase of the electrode connection track length (from the simulation by FEM (Finite Element Method)).

**Figure 9:**
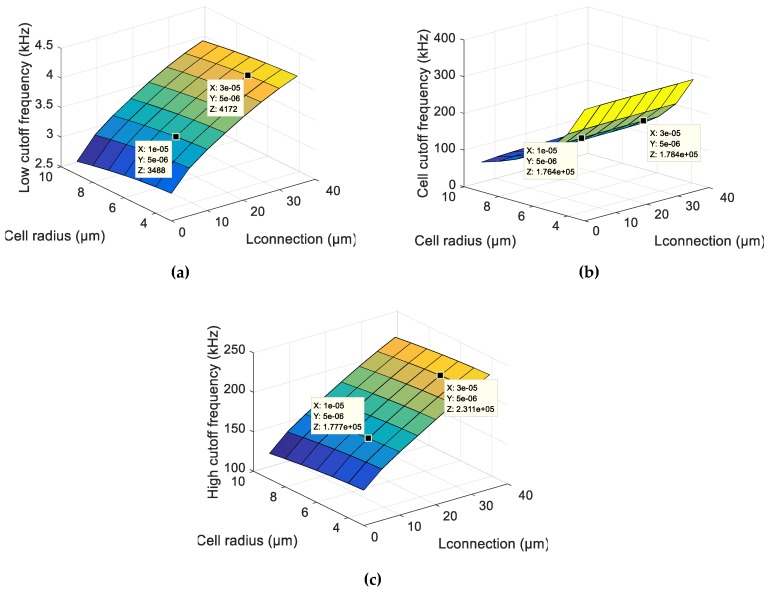
(**a**) Low cutoff frequency; (**b**) cell cutoff frequency; and (**c**) high cutoff frequency in the function of the connection length and cell radius from the numerical model.

To analyse the influence of electrode connection tracks on the cutoff frequencies, cutoff frequency graphs were established as a function of the electrode connection track length in contact with the solution and the radius of the cell (Figure 9). We can see that larger electrode connection track lengths led to higher cutoff frequencies. For a low cutoff frequency, as seen in Figure 9a, the increase of the surface and, consequently, the increase of the double-layer capacity decreases the impedance and, thus, increases the low cutoff frequency. For the high cutoff frequency seen in Figure 9c and the cell cutoff frequency seen in Figure 9b, this increase is because there is a rise in the charge transfer that causes a decrease in the impedance and, thus, an increase in the frequency.

## 4. Sensor Realization

Sensor realization is divided into two parts, the first one of which is the functional part of the electrodes on a glass substrate and the second of which is a microfluidic channel formed with polydimethylsiloxane (PDMS). The bonding of these two parts is done by a surface treatment of the PDMS with corona plasma. 

**Figure 10:**
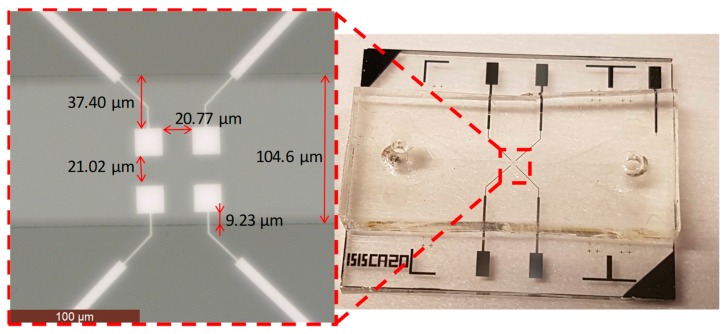
Image of the electrodes with their dimensions and spacing.

The dimensions of the electrodes and electrode connection tracks were chosen after the analysis of the simulations. The electrodes were 20 × 20 μm², and the electrode connection tracks were made as thin as possible with good levels of quality and repeatability for completion of the lithography step available in our laboratory (2 μm). In order to reduce the parasitic capacitance effects, the electrode connection track distances were increased. In Figure 10, the electrodes with their dimensions are shown. The channel has been shifted in one case to have a connection pair more exposed to the solution than in the other one.

### 4.1. Functional Part

**Figure 11:**
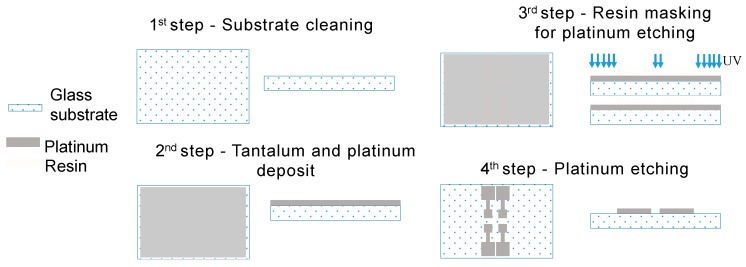
Realization process for the functional part.

The realization process of the functional part is shown in Figure 11. The glass substrates are cleaned with detergent, acetone, and isopropanol to remove all organic and inorganic materials. To have a good adhesion of the platinum to the substrate, tantalum was used as an intermediate. The etching of the metal was performed with an ion beam process.

### 4.2. Microfluidic Part

**Figure 12:**
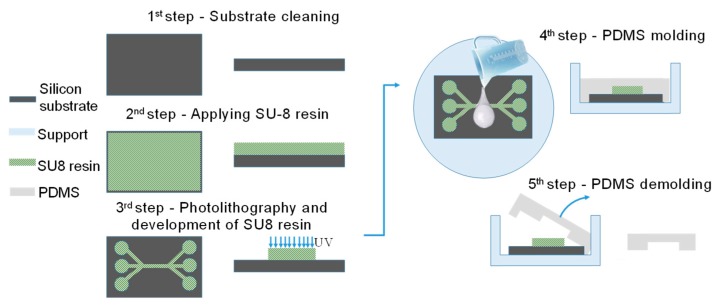
Realization process for the microfluidic part.

Figure 12 shows a schematic of microfluidic channel realization. This part is made using the organomineral polydimethylsiloxane polymer (PDMS) and a mould. The mould was made with the SU-8 photopositive thick resin on a silica substrate. Here, PDMS as a liquid form is mixed with a crosslinking agent and poured into a microstructural mould. 

## 5. Experiments

### 5.1. Samples

To show the functionality of the sensor in detecting a unit cell between the electrodes and to present the effects of the connection tracks on the normalized impedance variation, microbeads measuring 10 μm in diameter (Polybead® Black Dyed Microspheres, Polysciences Europe GmbH, Germany) were used. The microbeads were diluted in tap water (proportion of 1:10) to establish a laboratory situation that was closer to being like an on-site measurement situation. The tap water was electrically characterized in our laboratory and was found to have an electrical conductivity of 300 μS/cm. A standard solution of 1413 µS/cm was used then to characterize the sensor. 

### 5.2. Measurement Setup

**Figure 13:**
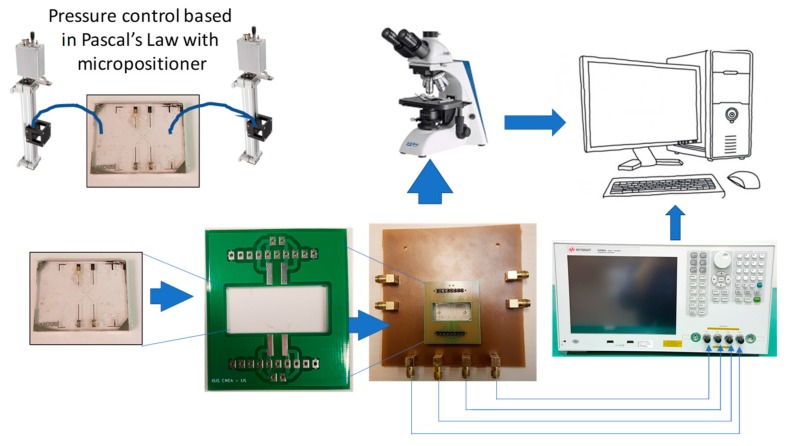
Schematic of the measurement setup.

An image of the measurement setup is shown in Figure 13. The measurement setup was composed of a micropositioner to place the sample on the electrode, a microscope with a CMOS camera to record the location of the sample on the electrode, a Keysight E4990A impedance analyser (Keysight Technologies, Santa Rosa, CA, USA) to measure the impedance spectrum, and a computer to record the sample images and the data. A liquid dielectric characterization kit (Keysight 16452A Liquid Test Fixture; Keysight Technologies, Santa Rosa, CA, USA) was also used with the impedance analyser to characterize the tap water.

### 5.3. Practical Results

Experimental measurements were completed to show the electrode connection track effects on the normalized impedance variation and on the cutoff frequencies. For this, we completed the measurements between the parallel electrodes A and B (electrode A with a high electrical level and B with a low electrical level) and between the electrodes C and D (Figure 15) as well as between the opposite electrodes A and C and electrodes B and D. The first measurements were done with the sensor empty (without solution on the electrodes) in order to extract the parasitic capacitance values. As the capacitance of the empty electrodes is much smaller than the parasitic capacitance, it can be said that the capacitance measured at an empty measure is the sum of all the parasitic capacitances, i.e., *C_subp_*, *C_p_*, and *C_dlp_*. The parasitic capacitance value for electrode pair A and B is 85 pF and for C and D is 82 pF. For the opposite electrodes, the parasitic capacitances are 78 pF for electrode pair A and C and 79 pF for electrode pair B and D.

**Figure 14:**
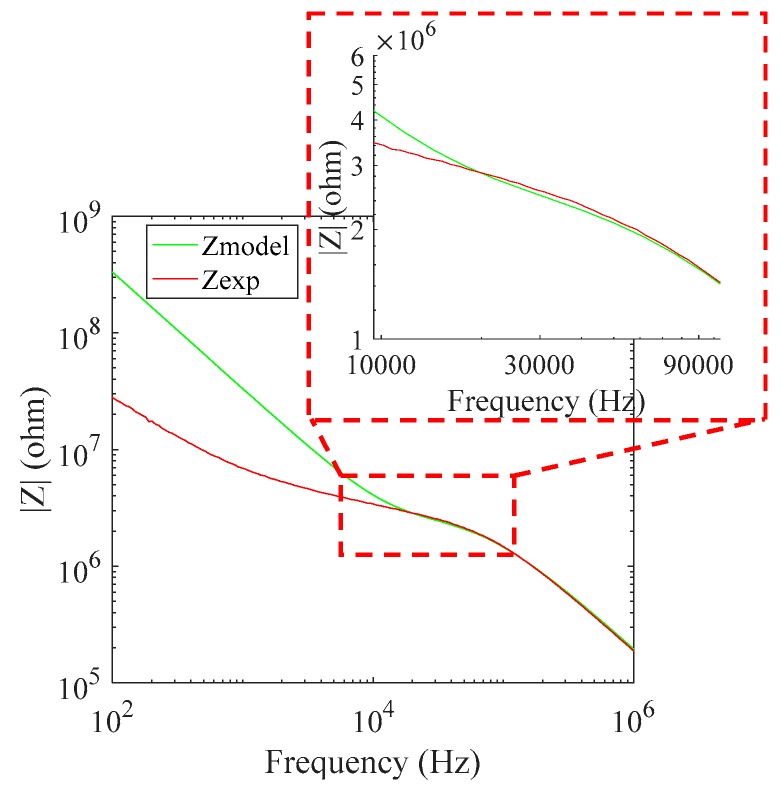
The impedance spectrum of the electrical model (green) and the experimental results (red) of 20 × 20 µm² electrodes with 10 µm electrode connection track lengths in solution.

In the continuity of the characterization of the sensor, measurements were made with the characterized tap water and a standard solution of 1413 µS/cm, and we extracted the value of the shape factor for the electrode pairs (K_AB_ = 60,000, K_CD_ = 62,000, K_AC_ = 61,000, and K_BD_ = 61,500). With the values extracted from the practical results, we performed the simulation of the electric model, as shown in Figure 14. In the low-frequency situation, we found that the experimental curve (red) is different from the model graph (green). However, this does not matter because we extracted the parameters from the plated frequency band in which the experimental curve and model curve were fitted. To promote understanding of the model, the effects of the interaction between the electrode and the solution were simplified to the double-layer capacitance; however, in reality, it has a diffusion effect, which can be modelled in a complex way by the Warburg element.

**Figure 15:**
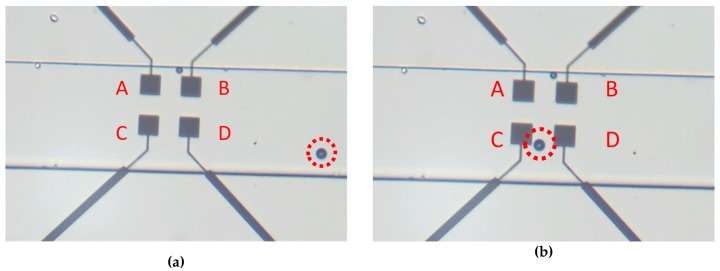
Microscope image of the electrodes (**a**) without the microbeads between the electrodes and (**b**) with a microbead between electrodes C and D.

To obtain the normalized impedance variation, the measurements were done with and without microbeads between the electrodes. For this, the solution with the microbeads was inserted into the pipes and pushed to the exit of the channel. All operations were monitored by the microscope, and when there were microbeads on the electrodes, the flow of the solution was stopped (decreasing the pressure variation in the pipes) so as to make the measurements of the microbeads static. The first measurement was done without any beads between the electrodes (Figure 15a) to establish the reference impedance, and then, the microbeads were moved to the electrodes to complete a second measurement with the microbeads located between the electrodes (Figure 15b). 

**Figure 16:**
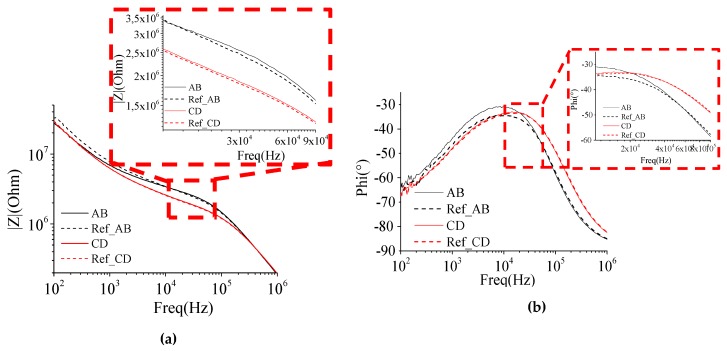
(**a**) The impedance spectrum and (**b**) the phase of the measurements between electrodes A and B (black) and between electrodes C and D (magenta).

The impedance spectra for the A and B and the C and D electrodes can be viewed in Figure 16. As the C and D electrode connection tracks were more exposed to the solution than the A and B electrode connection tracks, their impedance were smaller. This is because a larger electrode area means a larger shape factor and a smaller impedance.

The normalized impedance variation was calculated by the reference impedance (in 30 kHz) minus the impedance of the microbeads between the electrodes, and this variation was divided by the reference impedance. By doing this calculation, we obtained a normalized impedance variation from 3.7% to 4.2% for different measurements of A and B electrodes and from 1.8% to 2.1% for different measurements of C and D electrodes. For the opposite electrodes, the normalized impedance variation for different measurements of A and C electrodes is from 2.3% to 3% and for different measurements of B and D electrodes is from 2.2% to 2.8%. The difference of a ratio ×2 between the biggest and the smallest normalized impedance variation is due to the difference in the dimensions of the electrode connection track in contact with the solution that causes a leakage of the current. As the electrode connection track of the electrodes C and D have a big surface in contact with the solution, the normalized impedance variation for opposite electrodes (A and C, and B and D) is worse than that of the normalized impedance variation for parallel measures A and B. To standardize measurements between any electrode pair, we use an insulation layer of SiO_2_ on the connection tracks to isolate them electrically and to reduce their parasitic effects, as proposed in Reference [6].

The experimental low cutoff frequencies are very close to the analytical ones and the ones simulated by FEM. However, for the experimental high cutoff frequencies, there is a decrease that comes from the fact that the experimental parasitic capacitance is greater than those estimated by simulations. We see that the variation of the frequency band is negligible because it has just a displacement of the band.

## 6. Conclusions

To increase the limit of detection, it is necessary to reduce the electrode dimensions to the scale of the target biological cells. On the other hand, when the dimensions of the electrodes are reduced, undesired connection effects decrease the normalized impedance variation. To analyse these undesired connection effects, simulations using the finite element method were performed and a biosensor was fabricated and measured to confirm.

From the electrical model, the transition frequency equations (*F_low_*, *F_c_*, and *F_high_*) were found and, from these equations, the frequency in which the cell effects are exposed is known. From simulations and practical results, it can be seen that the larger the connection, the larger are the *F_low_* and *F_high_* cutoff frequencies but that the variation of the frequency band is negligible. 

The main effect of the electrode connection tracks is on the normalized impedance variation, in that we see an alteration of almost 50% between a pair of parallel electrodes with 9 μm of electrode connection track in contact with the solution and another pair of parallel electrodes with about 40 μm of electrode connection track in contact with the solution. Therefore, to reduce the effects of the connection on the normalized impedance variation and, thus, to have a better detection limit, we must reduce the width of the microchannel near to the electrode matrix dimension. Another possibility to reduce the connection effects on the normalized impedance variation is to use the opposite electrode connection track; however, when a matrix with at least 3 × 3 electrodes is used, the connection tracks for the central electrodes will be in parallel with at least another connection track.

The results here allowed us to see that the effects of the connections can reduce the normalized impedance variation up to 50% and that, in the case where the number of electrodes of a matrix increases, the electrodes more in the centre of the matrix will have more connections exposed to the solution and will have a lower normalized impedance variation. In perspectives, to solve this problem, we use an insulation layer of SiO_2_ on the connection tracks to isolate them electrically and to reduce their parasitic effects.

## References

[1] Wang R., Lum J., Callaway Z., Lin J., Bottje W., Li Y. (2015). A Label-Free Impedance Immunosensor Using Screen-Printed Interdigitated Electrodes and Magnetic Nanobeads for the Detection of E. coli O157:H7. Biosensors.

[2] Han S.I., Han K.H., Joo Y.D. (2013). An electrorotation technique for measuring the dielectric properties of cells with simultaneous use of negative quadrupolar dielectrophoresis and electrorotation. Analyst.

[3] Claudel J., Nadi M., Elmazria O., Kourtiche D. (2016). An electrical model optimization for single cell flow impedance spectroscopy. Int. J. Smart Sens. Intell. Syst..

[4] Rho J., Jang W., Hwang I., Lee D., Lee C.H., Chung T.D. (2018). Multiplex immunoassays using virus-tethered gold microspheres by DC impedance-based flow cytometry. Biosens. Bioelectron..

[5] Zhao Y., Zhao X., Chen D., Luo Y., Jiang M., Wei C., Long R., Yue W., Wang J., Chen J. (2014). Tumor cell characterization and classification based on cellular specific membrane capacitance and cytoplasm conductivity. Biosens. Bioelectron..

[6] De Araujo A.L.A., Claudel J., Nadi M., Kourtiche D. Detection and characterization of biological cells by impedance spectroscopy. Proceedings of the 2018 12th International Conference on Sensing Technology (ICST).

[7] Kokkinos C., Economou A., Prodromidis M.I., Prodromidis M. (2016). (Mamas) Electrochemical immunosensors: Critical survey of different architectures and transduction strategies. TrAC Trends Anal. Chem..

[8] Zhao Y., Chen D., Luo Y., Li H., Deng B., Huang S.-B., Chiu T.-K., Wu M.-H., Long R., Hu H. (2013). A microfluidic system for cell type classification based on cellular size-independent electrical properties. Lab Chip.

[9] Du E., Ha S., Diez-Silva M., Dao M., Suresh S., Chandrakasan A.P. (2013). Electric impedance microflow cytometry for characterization of cell disease states. Lab Chip.

[10] Asphahani F., Zhang M. (2007). Cellular Impedance Biosensors for Drug Screening and Toxin Detection. Anal..

[11] Stoneman M.R., Kosempa M., Gregory W.D., Gregory C.W., Marx J.J., Mikkelson W., Tjoe J., Raicu V. (2007). Correction of electrode polarization contributions to the dielectric properties of normal and cancerous breast tissues at audio/radiofrequencies. Phys. Med. Boil..

[12] Morgan H., Sun T., Holmes D., Gawad S., Green N.G. (2007). Single cell dielectric spectroscopy. J. Phys. D. Appl. Phys..

[13] Varshney M., Li Y., Srinivasan B., Tung S. (2007). A label-free, microfluidics and interdigitated array microelectrode-based impedance biosensor in combination with nanoparticles immunoseparation for detection of Escherichia coli O157:H7 in food samples. Sensors Actuators B: Chem..

[14] Ameri S.K., Singh P.K., Dokmeci M.R., Khademhosseini A., Xu Q., Sonkusale S.R. (2014). All electronic approach for high-throughput cell trapping and lysis with electrical impedance monitoring. Biosens. Bioelectron..

[15] Cheung K., Gawad S., Renaud P. (2005). Impedance spectroscopy flow cytometry: On-chip label-free cell differentiation. Cytom. Part A.

[16] Sabuncu A.C., Stacey M., Craviso G.L., Semenova N., Vernier P.T., Leblanc N., Chatterjee I., Zaklit J. (2018). Dielectric properties of isolated adrenal chromaffin cells determined by microfluidic impedance spectroscopy. Bioelectrochemistry.

[17] Ibrahim M., Claudel J., Kourtiche D., Nadi M. (2013). Geometric parameters optimization of planar interdigitated electrodes for bioimpedance spectroscopy. J. Electr. Bioimpedance.

[18] Couniot N., Afzalian A., Van Overstraeten-Schlögel N., Francis L., Flandre D. (2015). Capacitive biosensing of bacterial cells: Analytical model and numerical simulations. Sensors Actuators B: Chem..

